# Competing Neural Responses for Auditory and Visual Decisions

**DOI:** 10.1371/journal.pone.0000320

**Published:** 2007-03-28

**Authors:** Grit Hein, Arjen Alink, Andreas Kleinschmidt, Notger G. Müller

**Affiliations:** 1 Cognitive Neurology Unit, Johann Wolfgang Goethe-University, Frankfurt am Main, Germany; 2 Brain Imaging Center, Frankfurt am Main, Germany; 3 Helen Wills Neuroscience Institute, University of California, Berkeley, United States of America; 4 Department of Neurophysiology, Max Planck Institute for Brain Research, Frankfurt am Main, Germany; 5 Institut National de la Santé et de la Recherche Médicale (INSERM), Unité 562, Gif-sur-Yvette, France; 6 Commissariat à l'énergie atomique (CEA), Life Sciences Division (DSV), I2BM, NeuroSpin, Gif-sur-Yvette, France; University of Minnesota, United States of America

## Abstract

Why is it hard to divide attention between dissimilar activities, such as reading and listening to a conversation? We used functional magnetic resonance imaging (fMRI) to study interference between simple auditory and visual decisions, independently of motor competition. Overlapping activity for auditory and visual tasks performed in isolation was found in lateral prefrontal regions, middle temporal cortex and parietal cortex. When the visual stimulus occurred during the processing of the tone, its activation in prefrontal and middle temporal cortex was suppressed. Additionally, reduced activity was seen in modality-specific visual cortex. These results paralleled impaired awareness of the visual event. Even without competing motor responses, a simple auditory decision interferes with visual processing on different neural levels, including prefrontal cortex, middle temporal cortex and visual regions.

## Introduction

Why is our attentional capacity so limited? For example, most of us know situations in which we are trying to read while other people talk. Often, we will find ourselves reading the same paragraph over and over again, because auditory processing interfered with the processing of the visual input. Interference is often worst if simultaneous activities are very similar, suggesting competing demands on shared processing or neural systems [1], [2]. Behavioural indicators of interference such as increase of reaction time or error rate, however, are even seen for tasks with little common content [3]–[5]. These behavioural results suggest that simultaneous activities must compete at multiple levels, some more local and important when tasks are closely similar, some more global and contributing to interference even when tasks are very different.

One recent proposal of this sort is the “global neuronal workspace” model of Dehaene and colleagues [6], [7]. According to this model, conscious events depend on coactivation of local processors, e.g. modality-specific regions of visual or auditory cortex, and neurons in a global neuronal workspace. This workspace provides a flexible, selective representation or working memory of task-relevant cognitive content (see also refs. 8–10). Tentatively, the workspace might be localized in regions of frontal and parietal cortex, whose activation is associated with a wide range of different cognitive demands [11], [12]. Here we use fMRI to examine the basis for interference between simple auditory and visual decisions. One assumption is that these very different tasks recruit similar “global neuronal workspace” in frontoparietal regions. Modulation of activity in commonly recruited frontal and parietal areas could then be a basis for interference when tasks are performed together. Alternatively, different tasks might recruit common frontoparietal regions, but compete more strongly for workspace in the frontal component of the network. Supporting this assumption, a recent review showed overlapping activity for very different tasks in frontal cortex, whereas parietal activity correlated more strongly to processing of similar, i.e., visual tasks [13]. Moreover, based on the “global neuronal workspace” model, modulation of activity in frontal or frontoparietal workspace neurons should cause modulation of activity in co-activated local processors, e.g., visual or auditory cortex.

Previously, interference between auditory and visual processing has been investigated with auditory and visual tasks, which both required speeded responses [14]–[21]. If the tasks were presented closely together in time, reaction time for the second response increased, indicating interference between the processing of the tasks. The most consistent result was that this increase in reaction time correlated to increased dorsolateral prefrontal activity [reviewed in 13; but see 21]. However, given that subjects had to perform two responses under time pressure, this result might reflect motor competition rather than interference between auditory and visual processing. Even if different response modalities were used [20], subjects had to give an immediate response to the first task, which might have interfered with the processing of the second task. Other recent studies investigated visual and auditory tasks without speeded responses, but also without behavioural evidence for interference between visual and auditory processing [22]–[25]. Some used a design in which either the auditory or the visual modality was relevant [22], [23], [25]. Subjects did very well in identifying and encoding events in the relevant modality, which correlated to increased activity in the respective sensory cortices. Activity in auditory and visual cortex was increased independently of the relevant modality if the inputs were reliably paired [25]. If both modalities were relevant, subjects showed increased dorsolateral prefrontal activity, but still equally good performance in both tasks [24].

**Figure 1:**
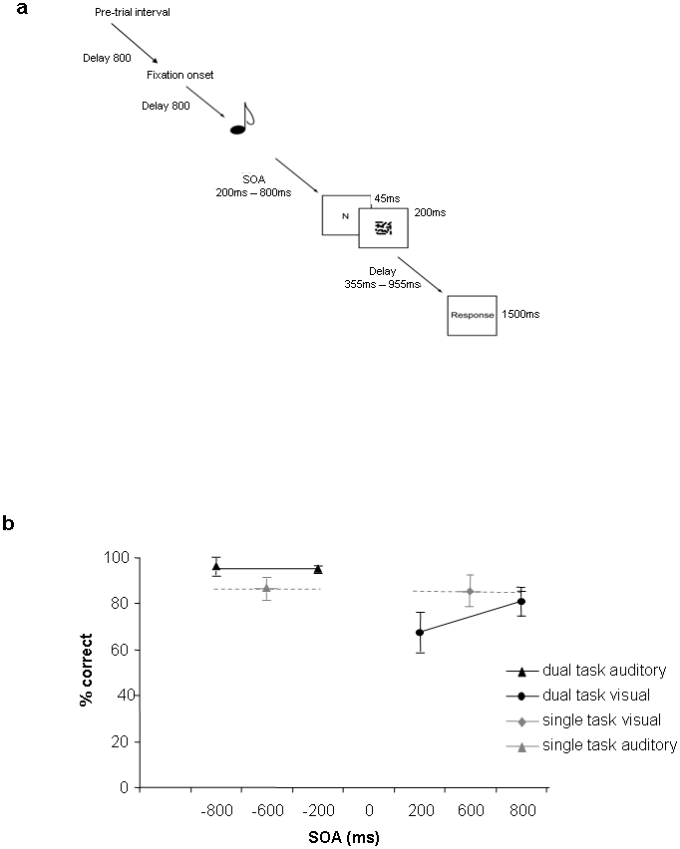
**a.** Example dual task trial. SOA from tone to letter was 200 or 800 ms. Single task tones and letters (here not shown) occurred at a fixed time (200 ms) from trial onset. b. Behavioural data from fMRI session. For dual task trials, SOA indicates interval between tone and letter, with tone data plotted at negative SOA and letter data at positive SOA. For comparison, single tone and letter data are plotted at a notional SOA. Letter data are for target trials. Error bars indicate standard errors.

The novelty of our experiment is that we used a paradigm which (A) provided clear behavioural evidence for interference between auditory and visual processing, but (B) was not confounded by effects of motor competition. We made use of the well-known “attentional blink” occurring when a brief visual target is presented during processing of an auditory tone [5]. Under these conditions, visual identification is typically impaired for a period of several hundred ms following tone onset. In our version of the experiment (**Figure 1a**), a keypress was made to show which of three alternative tones had been presented. At varying intervals (stimulus onset asynchrony or SOA) after the tone, a letter was flashed briefly on a computer screen. The subjects decided whether this letter was a specific target (“N”), if so making a further keypress. The experiment also included control trials with just a single task, auditory or visual, which occurred at a fixed temporal position shortly after trial onset. Subjects were told to take their time over the visual and the auditory decision and to give unspeeded responses at the end of the trial.

Based on the “global neuronal workspace” model we test the following predictions. Firstly, events as dissimilar as visual and auditory decisions should recruit similar “global neuronal workspace”, probably located in frontoparietal cortex. Secondly, if they are performed together, auditory and visual decisions should compete for frontoparietal “global workspace”, indicated by modulation of frontoparietal activity. Alternatively, competition for frontal “workspace” might be stronger than in parietal cortex [13], correlating to stronger modulation of frontal activity as compared to parietal activation. Thirdly, modulation of activity in frontal or frontoparietal “global workspace” should modulate activity in co-activated local processors, reflected by modulation of activity in visual or auditory cortex.

## Results

Behavioural data are shown in **Figure 1b**. As expected, visual target detection was substantially impaired at short SOA (**Figure 1b**, black symbols), whereas single task accuracy was uniformly high (**Figure 1b**, grey symbols). In dual task trials, letter performance was significantly impaired if the letter was presented shortly after the tone, compared to the long SOA condition and compared to single letter trials (both p<0.02). The rate of false alarms, i.e., nontarget letters incorrectly identified as targets, was very low (dual task visual, SOA 200, <3.2%; dual task visual, SOA 800, <4.1%; single task_visual<6.2%). Tone performance in dual task trials was better than in single auditory trials, p<0.03. It is possible that subjects dedicated more attention to the tone response in dual task trials than in single auditory trials, which could account for the difference in performance between the conditions. Such potential differences in arousal between single and dual task trials did not affect our fMRI analysis, because we only contrasted dual task trials with short and long SOA.

**Figure 2:**
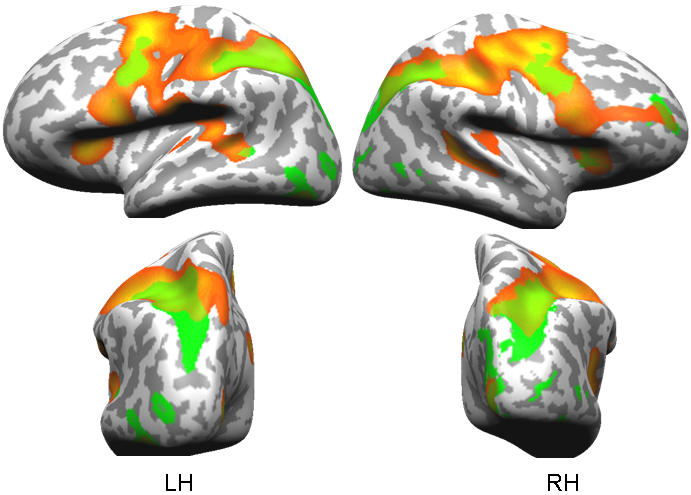
Significant activity (q(FDR)<.001 for both activation maps) for the contrast between single visual trials (orange) and single auditory trials (green) trials versus ‘null trials’ baseline. LH – left hemisphere, RH – right hemisphere. A list of cortical regions commonly activated by single visual trials and single auditory trials is provided in Table 1.

For fMRI data, our first prediction was that auditory and visual tasks would show overlapping activation in frontal and parietal cortex. Our results confirmed this prediction. When compared with “null” trials, containing no stimulus or decision, single task auditory and single task visual trials both showed significant activation in left and right dorsolateral prefrontal and posterior parietal regions. We further found overlapping activity in left middle temporal gyrus, which has been characterised as multimodal region in previous studies [26], [27] (**Figure 2**, **Table 1**).

**Table 1:** Regions commonly activated by single task visual and single task auditory trials, based on a [single task visual – null] ∩ [single task auditory – null] conjunction analysis.

		Talairach coordinates				
Cortical area	BA	x	y	z	Mean t value	voxel
Right insula	13	39	12	10	4.13	681
Right precentral gyrus	6	39	0	33	4.6	7844
Left precentral gyrus	6	−43	−3	36	4.8	8511
Left middle frontal gyrus	6	−17	−2	59	4.14	619
Right middle frontal gyrus	10	33	42	22	4.2	1751
Right superior frontal gyrus	6	26	−59	36	5.0	8879
Left posterior cingulate	23	−1	−25	21	4.18	878
Left superior parietal lobe	7	−30	−54	38	5.23	13490
Right parietal lobe	7	26	−59	36	5.0	8879
Left middle temporal gyrus	21	−50	−47	5	4.2	698

The Talairach coordinates indicate the center of mass significantly activated (p<0.001; FDR corrected). BA – Broadman area.

**Figure 3:**
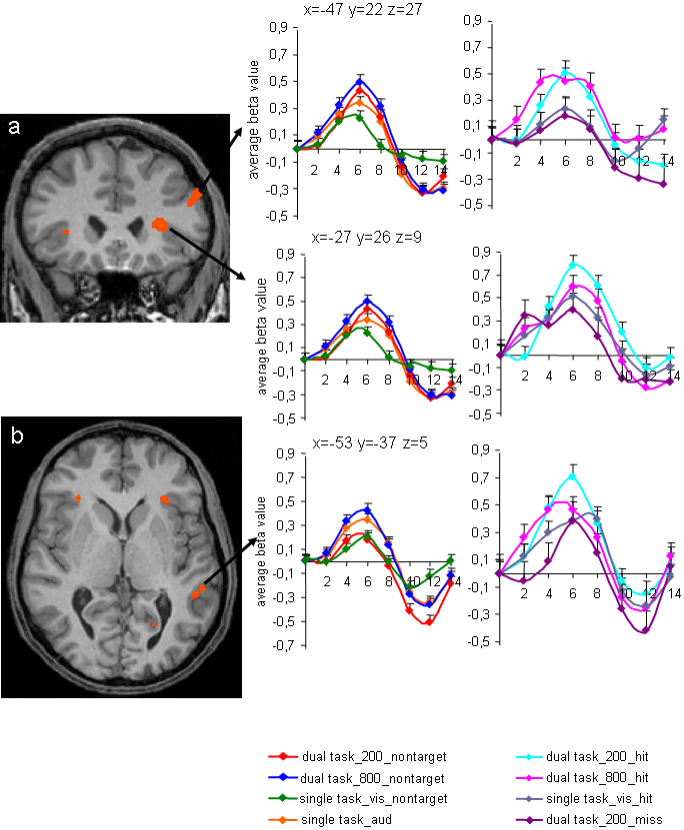
Significant activity (p<.03, uncorrected; voxel threshold>60) for the whole brain contrast between dual task trials with long SOA and dual task trials with short SOA in a) frontal cortex and b) middle temporal gyrus. Details are provided in Table 2. Time courses of activity were extracted for dual task trials with short and long SOA and nontarget letters (dual task_200_nontarget; dual task_800_nontarget), dual task trials with short and long SOA and successfully identified letter targets (dual task_200_hit; dual task_800_hit), single visual trials with nontargets and successfully identified letter targets (single task_vis_nontarget, single task_vis_hit), single auditory trials (single task_aud) and dual task trials with short SOA and letter target misses (dual task_200_miss), in contrast to the null trial activation baseline. Error bars indicate standard errors.

Our second prediction concerned competition in dual task trials. One assumption was that behavioural interference between auditory and visual tasks at short SOA modulates activity of frontal and parietal workspace neurons. Alternatively, interference between dissimilar tasks was expected to be stronger in the frontal cortex, in line with a recent review [13]. To eliminate any motor component in the visual task, the whole brain analysis was based on nontarget trials (see Methods). The contrast between dual task trials with long and short SOA showed focused activity in frontal cortex (**Figure 3a**, left panel; **Table 2**). Foci of frontal activity were located bilaterally in ventrolateral prefrontal cortex (VLPFC) around the insula and in left middle frontal gyrus (MFG). Reduction of dual task activity in VLPFC and MFG paralleled subjects' failure to detect the letter target (all p<0.05; **Figure 3a**, right panel). In case of successful letter target detection, activity for dual task trials with short SOA was comparable or even slightly higher than in dual task trials with long SOA (all p>0.4). Accordingly, it is unlikely that our frontal effects are based on higher task switching costs at short SOA, because those would predict a similar impact of SOA on dual task activity in dual task trials with letter targets and nontargets.

**Table 2:** Significant activity for the whole brain contrast between dual task trials with long SOA and dual task trials with short SOA.

		Talairach coordinates				
Cortical area	BA	x	y	z	Mean t value	voxel
Right insula	13	31	22	6	2.24	102
Left insula	13	−27	26	9	2.57	1065
Left middle frontal gyrus	46	−46	21	26	2.26	309
Left middle temporal gyrus	22	−53	−38	4	2.18	74
Left occipital lobe/lingual gyrus	19	−23	−62	0	2.19	63

The Talairach coordinates indicate the center of significantly activated (p<0.03) clusters with a cluster size of at least 60 voxels. BA – Broadman area.

Resembling the pattern of results in frontal cortex, we further found effects in left middle temporal gyrus (MTG; **Figure 3b**, left panel; **Table 2**). MTG showed reduced activation for dual task trials in which subjects missed the letter target (p<0.03; **Figure 3b**, right panel). The similarity of results in frontal regions and MTG is in line with primate data, showing strong anatomical connections between prefrontal cortex and middle temporal regions [28], [29].

Our first findings showed that interference between auditory and visual decisions correlates to reduced activity in frontal regions and multimodal middle temporal cortex. This supports the assumption that auditory and visual decisions compete for frontal “workspace”, even without competing motor responses.

**Figure 4:**
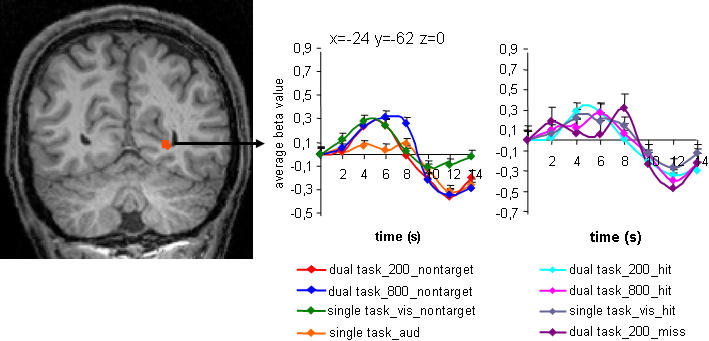
Results of the whole brain contrasts between dual task trials with long SOA and short SOA in modality specific cortices. Significant activity (p<.03, uncorrected; voxel threshold>60) was found in modality-specific visual cortex (see also Table 2), but not in auditory regions. Time courses show activity for dual task trials with short and long SOA and nontarget letters (dual task_200_nontarget; dual task_800_nontarget), dual task trials with short and long SOA and successfully identified letter targets (dual task_200_hit; dual task_800_hit), single visual trials with nontargets and successfully identified letter targets (single task_vis_nontarget, single task_vis_hit), single auditory trials (single task_aud) and dual task trials with short SOA and letter target misses (dual task_200_miss), in contrast to the null trial activation baseline. Error bars indicate standard errors.

A third question concerns responses in modality-specific cortex. What should we expect for such contrasts? One possibility is that, for modality-specific systems, there is always parallel processing. This would predict that there is no difference in activity between dual task trials with short and long SOA in auditory and visual cortex. In line with other proposals [8]–[10] however, the global workspace model proposes that attention involves mutual support between local, modality-specific processors and frontal workspace neurons. If responses to a visual target are suppressed in the global workspace, this model predicts that support will also be lost in modality-specific cortex. The results are in line with this prediction. As in frontal cortex and MTG, activity in visual cortex was stronger for dual task trials with long SOA as compared to short SOA (**Figure 4**, left panel). The difference in activity for dual task trials with short SOA and target hits versus target misses was not significant (p>0.5), but there were differences in the shape of the BOLD response (**Figure 4**, right panel). Unlike dual task trials with target hits, dual task trials with short SOA and target misses evoked an early small peak of activation and a second, higher peak, which was delayed compared to all other conditions. The contrast between dual task trials with long and short SOA did not show any effect in auditory regions, indicating preserved neural response to the auditory target.

**Figure 5:**
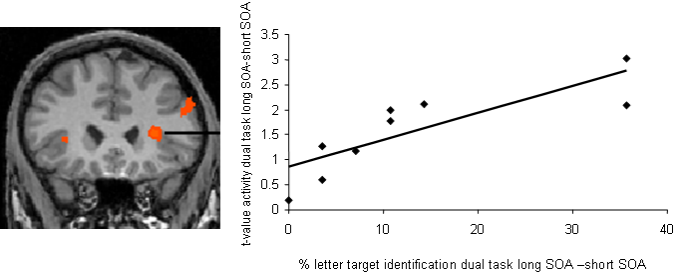
Significant correlation between suppression of VLPFC activity at short SOA and the strength of interference between visual and auditory processing in individual subjects. Strength of interference was calculated as the difference between the percentage of correctly identified letter targets at short and long SOA.

The suppression of activity in frontal regions, middle temporal cortex and visual cortex paralleled impaired awareness of the visual event, but is it correlated to the strength of interference between auditory and visual decisions in individual subjects? The strength of interference is indexed by the difference between correctly identified target letters in dual task trials with short and long SOA. We calculated this index for each subject (**Table 3**) and correlated it to the individual differences in dual task activity at short and long SOA in the VLPFC, MFG, MTG and visual regions found in the group analysis (**Figures 3** and **4**). The results showed that higher impairment of letter identification in dual task trials with short SOA correlated with stronger suppression of activity in VLPFC, r = 0.8, p<.01, (**Figure 5**). The impact of auditory processing on letter target detection varied across subjects (**Table 3**), which accounts for the moderate significance level of the frontal effects in the group analysis (**Figure 3**, p<0.03 uncorrected). The correlation analysis showed no significant results in any other region. VLPFC is known to be involved in controlled selection and retrieval [30], which is in line with the assumption that the global workspace provides working memory of task-relevant cognitive contents. According to our results, limitations in frontal global workspace differ among subjects. Subjects with reduced VLPFC activity at short SOA showed strong impairment in the second, i.e., visual decision, while still giving a correct response to the first, i.e., auditory stimulus. Possibly this indicates that in these subjects limited global workspace allowed the correct processing of only one, the first (auditory) decision, but not of a temporally overlapping second (visual decision). In order to correctly perform the first (auditory) decision, processing of the competing visual target is not supported, resulting in a decrease of VLPFC workspace activity. The result of increased interference with decreased frontal activity is in line with findings of aging and patient studies [10], [31].

**Table 3:** Impairment in letter detection in individual subjects, t and p values for the individual contrast between dual task trials with long and short SOA, and talairach coordinates of the ventrolateral prefrontal region in which this contrast was calculated.

				Tailairach coordinates		
Subject	% missed letters	p	T	x	y	z
OD	0	0.62	0.49	−27	26	9
AA	3,5	0.09	1.7	−27	26	9
AP	3,5	0.28	1.07	−27	26	9
HD	7,1	0.14	1.4	−27	26	9
HB	10,7	0.57	0.55	−27	26	9
SE	10,7	0.052	1.9	−27	26	9
CA	14,3	0.17	1.4	−27	26	9
LA*	35,7	0.0025	3.03	−39	22	24
SS	35,7	0.09	1.68	−27	26	9
Average (SD)				−28 (4)	25 (1)	10 (5)

* LA showed no BOLD activity in the group ROI (x = −27, y = 26, z = 9). Here, we conducted an individual whole brain contrast between dual task trials with short and long SOA, searched for the peak of activity closest to −27 26 9 and defined it as ROI. To test the robustness of the correlation, we additionally performed the correlation analysis without LA, i.e., based on only eight subjects. The results confirmed the findings of the correlation analysis with nine subjects, r = .7; p<.05.

For all, except one subject, we used the region in left VLPFC revealed previously by the group analysis.

## Discussion

Overall, our findings suggest a general view of neural processes underlying limited attentional capacity. As behavioural data show, there is often limited capacity even for attention to very different cognitive events. We predicted that this might correlate to modulation of activity of “global neuronal workspace” neurons and co-activated local processors in visual or auditory regions. Firstly, our data supported the assumption that different tasks such as auditory and visual decisions recruit similar frontoparietal “workspace”. Secondly, we tested how this global workspace activity is affected by interference between auditory and visual decisions. Our results showed that interference between unspeeded auditory and visual decisions modulates activity in the frontal, but not the parietal, component of the frontoparietal workspace. Parietal activity found for single visual and auditory trials (**Figure 2**) might reflect stimulus-response mapping in the individual task [32]–[34], which, however, did not interfere in our unspeeded experiment. A third assumption was that one function of workspace neurons may be to support related processing in more local systems [8]–[10]. The result should be a distributed reduction of stimulus- or task-related activity when attention is focused elsewhere. Our findings in visual modality-specific cortex support this assumption, probably reflecting down-regulating frontal control via middle temporal regions.

Based on our results, competition in the frontal component of a “global neural workspace” provides at least a part of the basis for limited attentional capacity for dissimilar tasks, independently of response conflict. Of course, workspace competition in comparable regions is also expected for more similar events, such as two visual targets, in line with recent studies of within-modality interference [35]–[38]. In addition to frontal effects, these studies with similar, i.e., visual tasks showed modulation of parietal activity, although they had unspeeded responses [36]–[38]. Based on these result it was suggested [13] that parietal activity is more strongly modulated by interference between visual events (see also refs. 39, 40) and less involved in competition between dissimilar tasks, in line with our results. Modulation of parietal activity reported in two studies with speeded auditory and visual tasks [15], [19] might reflect interference between stimulus-response mappings [32–34.]

The extent of modulation of workspace activity in ventrolateral prefrontal cortex predicted the strength of audio-visual interference in individual subjects. This result further supports the important role of the frontal workspace component in attentional limitation for dissimilar tasks, even without motor competition. It is known that the magnitude of attentional competition varies across healthy young subjects [41], [42]. However, these interindividual variations have been largely ignored. One previous fMRI experiment [41] and one recent electrophysiological study [42] investigated inter-individual differences in modality-specific interference. Feinstein et al. [41] showed increased activity in frontal regions and anterior cingulate for “good” subjects, which showed little behavioural interference between visual stimuli. Martens et al. [42] reported an earlier onset of the P3 component, associated with working memory updating, in subjects with little behavioural competition between visual targets. Moreover, “good” subjects showed significant frontal selection positivity (FSP) effects over the ventrolateral prefrontal cortex, in line with the outcome of our single subject analysis. It is an interesting issue for further studies to explore the nature of behavioural and neuronal differences in attentional limitations in the healthy young population. According to our study, individual differences in frontal workspace activity should predict individual differences in the ability to process the various sorts of information we are confronted with in daily life.

## Material and Methods

All experiments were conducted at the Brain Imaging Centre in Frankfurt, Germany. Participants were paid for participation in the study conducted in conformity with the Declaration of Helsinki and approved by the local ethics committee.

Subjects. Twelve subjects (seven female) participated in one fMRI session and a preceding behavioural training outside the scanner. All subjects reported normal hearing and sufficient vision. The reported data is based on nine subjects, because we had to discard three data sets because of high error rates (>50%) in the behavioural single tasks.

Stimuli. The auditory stimulus was one of three tones (500, 600, 700 Hz) and the visual stimulus was a masked letter. Tones were presented over headphones with a duration of 200 ms. Letters were upper case, presented in the centre of a computer monitor (behavioural sessions; letter height 2.6 deg), or projected onto a screen, which subjects viewed via a mirror mounted in the bore of the magnet (fMRI sessions; letter height 1.2 deg). Letters were flashed for 45 ms, and immediately followed by a 200 ms backward mask of jumbled contours (**Figure 1a**). One third of all letters were targets (the letter N). The remaining letters were nontargets, drawn from a set of 10 other consonants, requiring no response.

### Procedure

The experiment involved (a) dual task trials, with a tone followed by a masked letter; (b) single task auditory trials; (c) single task visual trials; (d) null trials with no stimulus. All trial types were mixed in each block. Because of the slow BOLD response, in an event-related design activity in a current trial can be influenced by activation evoked by the preceding trial. To control for effects of activation history, the order of trial presentation was ‘one back’ counterbalanced so that trials from each condition listed above were preceded equally often by all trial types for one trial back [43]. Each trial lasted 4.5 s (**Figure 1a**), with an additional empty interval of 800 ms separating one trial from the next. Trials began with onset of a fixation cross at screen centre. Tones, if presented, occurred 200 ms after trial onset; letters occurred randomly at 400 or 1000 ms from trial onset, giving tone-letter SOAs of 200 and 800 ms on dual task trials.

Subjects gave their responses during a 1.5 s response window at the end of the trial. This response period was marked by the word “Response” (in German), which appeared on all trials (even those with no letter) in the centre of the screen (**Figure 1a**). Responses were given on a four-key button box, using the index and middle finger of each hand (left middle - 500 Hz, left index - 600 Hz, right index - 700 Hz, right middle – target letter). There was a behavioural training before the fMRI experiment, including two practice blocks each for single task visual trials, single task auditory trials and dual task trials (each practice block 32 trials) with feedback, followed by one block without feedback which resembled the experimental blocks in the fMRI session. While lying in the scanner, subjects again performed 10 single task visual and single task auditory practice trials with feedback to get used to the button box. This practice period in the scanner was also used to adjust the volume of the tone such that it was in the range of comfortable hearing for the individual subject. The fMRI main experiment included four experimental blocks. There were 100 trials per block, including 20 single task auditory trials, 20 single task visual trials, 20 null trials and 40 dual task trials, 20 trials for each SOA. One third of single letter trials and dual task trials had target letter (5 trials per block each).

### MRI data acquisition

Subjects were scanned on a 3T Siemens Magnetom Allegra scanner with a standard head coil. A gradient-recalled echo-planar-imaging (EPI) sequence was used with the following parameters: 34 slices; TR, 2000 ms; TE, 30 ms; FOV, 192 mm; in-plane resolution, 3×3 mm^2^; slice thickness, 3 mm; gap thickness, 0.3 mm. 301 scans were acquired per run, including 2 dummy scans to allow T1 equilibration. To maximize the quality of the EPI images, we ran an additional 30s sequence before each run. In this sequence we used a point spread function to estimate the disturbance of the magnetic field. The parameters determined by this point spread function were then applied to correct the EPI images acquired in the following run [44]. After functional scanning, for each subject we acquired detailed anatomical images using a magnetization-prepared rapid-acquisition gradient-echo (MP-RAGE) sequence (TR = 2300 ms, TE = 3.49 ms, FA = 12°, matrix = 256×256, voxel size 1.0×1.0×1.0 mm^3^).

### Data analysis

One third of trials with target letters (in total 40 dual task trials, 20 per SOA and 20 single task visual trials per session) entered the analysis of the behavioural data collected during fMRI scanning. The major part of the fMRI analyses (all except data shown in the right panel of Figure 3 and Figure 4) was based on the other two third of nontarget trials with maximal one correct (tone) response (in total 120 dual task trials, 60 per SOA, and 60 single task visual trials per session). This allowed us to eliminate response-related conflicts, which might occur between planning and execution of two successive motor responses even in our unspeeded design. Only correct trials were included in the fMRI analysis. Behavioural data was analyzed with SPSS software. Performance scores were assessed for auditory and visual targets in single and dual task trials, for the latter independently of the order of response. For dual task trials, mean accuracy scores were computed for each SOA. Calculation of letter target performance scores was based on dual task trials with a correct tone response. Mean accuracy scores were then submitted to paired t-tests (dual task short versus long SOA; single versus dual task performance).

All fMRI data were processed and analysed using Brain Voyager QX software (Brain innovation, Maastricht, The Netherlands). Standard pre-processing was conducted comprising three-dimensional motion correction using the Levenberg-Marquardt algorithm, linear-trend removal and temporal high-pass filtering at 0.0054 Hz and slice timing correction. The pre-processed data was then normalized and analysed with a deconvolution approach [45], providing whole brain maps and time courses of activity. Here, we estimated the effect size for each condition in 10 time bins of 2s each, whereby the first time bin represented the onset of the trial. In contrast to conventional imaging analyses, a deconvolution approach makes no assumption about the shape of the Hemodynamic Response Function (HRF) and takes into account differences in latencies of activation peaks and amplitude across different brain regions. Time courses of activity (**Figures 3** and **4**) show group-averaged beta values in the clusters of activation in seven of the ten time bins, covering a time window of fourteen seconds after trial onset. There were very few letter target misses in dual task trials with long SOA and single task visual trials. Therefore we abstained from extracting time courses of activity for these conditions. Statistical analysis (paired t-tests) between conditions was performed over the three time bins corresponding to the peak of the BOLD response [35]. Before the statistical analysis, these time bins were determined by collapsing all conditions together. Based on this, statistical analyses were conducted on averaged beta values in time bins 2, 3 and 4, corresponding to a time interval of four to eight seconds after trial onset. Whole brain activation maps were projected on the inflated cortical sheet of one subject or on a template brain normalized in Talairach space. They are based on normalized data analysed in a fixed-effects model. Significance thresholds are reported in the corresponding figure caption. Significant activations revealed in the group analysis were used as regions of interests (ROIs) for single subject correlation analyses. Here, we performed a two-tailed Pearson correlation analysis between the individual subjects' differences between the percentage of correctly identified letter targets at short and long SOA and individual p-values for the [dual task_800 versus dual task_200] contrast. To extract these p-values for each subject, we conducted individual ROI-based GLMs. P and t-values of the individual GLMs are reported in Table 3. We chose this approach rather then differences between percent signal change, because it takes into account the variability between effect sizes in the ten time bins per condition within each subject.
